# Complete Genome Sequence of Mycoplasma bovis Strain KG4397, Isolated from Cattle in Japan

**DOI:** 10.1128/MRA.00838-19

**Published:** 2019-10-03

**Authors:** Misako Morimoto, Tsuyoshi Kenri, Takashi Ohmori, Kaho Teshima, Kazumoto Shibuya, Chihiro Sasakawa, Masato Suzuki

**Affiliations:** a Nippon Institute for Biological Science, Ome, Tokyo, Japan; b Department of Bacteriology II, National Institute of Infectious Diseases, Musashimurayama, Tokyo, Japan; c Medical Mycology Research Center, Chiba University, Chiba, Japan; d Antimicrobial Resistance Research Center, National Institute of Infectious Diseases, Higashimurayama, Tokyo, Japan; University of Arizona

## Abstract

Mycoplasma bovis is a major bacterial pathogen that causes pneumonia, mastitis, and arthritis in cattle. In this study, we performed whole-genome sequencing of an M. bovis strain isolated in Japan for the first time and announce the complete genome sequence of strain KG4397, which caused respiratory diseases in cattle in 2012.

## ANNOUNCEMENT

Mycoplasma species belong to the family Mycoplasmataceae in the class Mollicutes. Mycoplasma bovis, belonging to the hominis group of *Mycoplasma*, is a major bacterial pathogen causing pneumonia, mastitis, and arthritis in cattle and is responsible for substantial economic losses to the cattle industry worldwide (1). Virulence-associated factors of M. bovis, including lipoproteins and secreted proteins, might contribute to bacterial adhesion and invasion into host cells and to subsequent survival and dissemination within hosts (2).

Here, we announce the complete genome sequence of M. bovis strain KG4397, which caused respiratory diseases in cattle on a farm in Kagoshima, Japan, in 2012. Strain KG439 was originally isolated from the nasal cavity of cattle by using swabs, was grown on pleuropneumonia-like organism (PPLO) agar (Difco), and was stocked after single-colony isolation. Antimicrobial susceptibility testing of strain KG4397 was performed using dry plate DP34 (Eiken Chemical), and the MICs to the 14-member macrolide clindamycin (CLDM) and the 15-member macrolide azithromycin (AZM) were relatively high (0.25 and 1.0 μg/ml, respectively). Consistent with this, Mycoplasma hominis, also belonging to the hominis group, was reported to be inherently resistant to CLDM and AZM (3).

Genomic DNA of strain KG439 grown on PPLO agar was extracted using NucleoBond AXG 500 and buffer set III (TaKaRa). Whole-genome sequencing of strain KG4397 was performed using the PacBio RS II system (Pacific Biosciences) with DNA sequencing reagent kit 4.0 v2 and SMRT Cell v3 and using the MiniSeq system (Illumina) with a high-output reagent kit (300 cycles). The library for PacBio sequencing (insert size of around 20 kbp) was prepared using the SMRTbell template prep kit v1.0, and the library for Illumina sequencing (insert size, 500 to 900 bp) was prepared using a Nextera XT DNA library prep kit. PacBio reads were base called using the SMRT Analysis Software v2.3.0, with default parameters (Pacific Biosciences), and were assembled *de novo* using Canu v1.7, with default parameters (4). The overlap region in the assembled contig was detected by a genome-scale sequence comparison using LAST (http://last.cbrc.jp) and was trimmed manually. Illumina paired-end reads were mapped onto the resulting circular chromosome, and sequencing errors were corrected by extracting the consensus of the mapped reads using CLC Genomics Workbench v11.0.1 (Qiagen).

**FIG 1:**
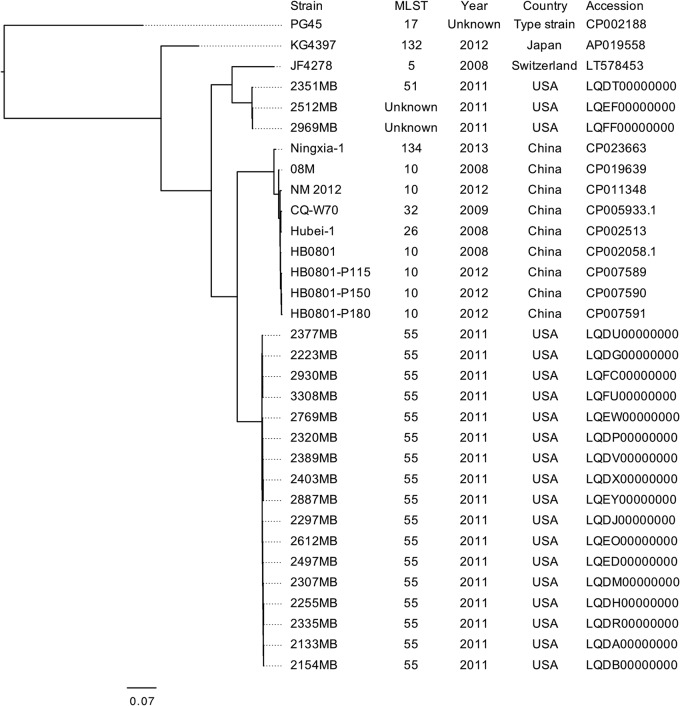
A genome-wide SNP-based phylogenetic analysis of M. bovis. The phylogenetic tree shows the strain names, sequence types from MLST analysis, isolation years, and isolation countries. The DDBJ/EMBL/GenBank accession numbers of the genome sequences of strains PG45, KG4397, JF4278, 2351MB, 2512MB, 2969MB, Ningxia-1, 08M, NM 2012, CQ-W70, Hubei-1, HB0801, HB0801-P115, HB0801-P150, HB0801-P180, 2377MB, 2223MB, 2930MB, 3308MB, 2769MB, 2320MB, 2389MB, 2403MB, 2887MB, 2297MB, 2612MB, 2497MB, 2307MB, 2255MB, 2335MB, 2133MB, and 2154MB are CP002188, AP019558, LT578453, LQDT00000000, LQEF00000000, LQFF00000000, CP023663, CP019639, CP011348, CP005933, CP002513, CP002058, CP007589, CP007590, CP007591, LQDU00000000, LQDG00000000, LQFC00000000, LQFU00000000, LQEW00000000, LQDP00000000, LQDV00000000, LQDX00000000, LQEY00000000, LQDJ00000000, LQEO00000000, LQED00000000, LQDM00000000, LQDH00000000, LQDR00000000, LQDA00000000, and LQDB00000000, respectively. The scale bar represents the number of estimated changes per position.

The compete genome sequence of strain KG4397 was constructed as a total sequence of 1,089,241 bp, with a mean G+C content of 29.3%. A total of 1,692 coding DNA sequences (CDSs) and 5 rRNAs, including two sets of 16S rRNAs, were annotated by the DDBJ Fast Annotation and Submission Tool (DFAST) server (5). The 16S rRNA sequences of strain KG4397 almost matched those of the M. bovis type strain PG45 (accession number CP002188; 99.8 to 99.9% similarity) in the BLAST analysis. Strain KG4397 belonged to a novel sequence type (ST), ST132, by multilocus sequence typing (MLST) in the PubMLST database (https://pubmlst.org), and CDSs of strain KG4397 encoded potential virulence-associated factors, such as liproproteins and secreted proteins. Publicly available genome sequences of M. bovis and their metainformation were collected from the PATRIC database (6), and a genome-wide single-nucleotide polymorphism (SNP)-based phylogenetic analysis of M. bovis with PG45 as a reference genome using CSI Phylogeny v1.4 (7), with default parameters, showed that strain KG4397 was classified into a genetic lineage of M. bovis distinct from that of strains isolated in other countries, such as the United States and China (Fig. 1).

In this study, we performed whole-genome sequencing of M. bovis, isolated in Japan, for the first time. Comparative genomic analysis with M. bovis isolated in other countries and regions will facilitate further comprehensive genomic epidemiological analysis, thus expanding our understanding of virulence and antimicrobial resistance phenotypes of M. bovis and leading to drug and vaccine development for M. bovis-associated infectious diseases in livestock.

### Data availability.

The complete genome sequence and raw sequencing data of M. bovis strain KG4397 have been deposited at DDBJ/EMBL/GenBank under the accession number PRJDB8066.
